# Editorial: Exploring the dynamics of tissue flexibility: molecular changes and their implications for metabolic disorders

**DOI:** 10.3389/fendo.2025.1757593

**Published:** 2025-12-16

**Authors:** Lynda Bourebaba

**Affiliations:** Department of Experimental Biology, Institute of Biology, Faculty of Biology and Animal Science, Wrocław University of Environmental and Life Sciences, Wrocław, Poland

**Keywords:** adipose tissue, cardiometabolic health, fibrosis, metabolic flexibility, metaflammation, mitochondria, organ crosstalk, skeletal muscle

## Background

Metabolic flexibility (MetFlex) reflects the dynamic capacity of the body to adapt fuel utilization according to fluctuating energy demands and nutrient availability, playing a fundamental role in maintaining systemic energy balance. This process relies on a tightly regulated network involving hormonal signals, enzyme activities, and intricate cellular signalling pathways that orchestrate nutrient sensing and substrate switching. Adipose tissue, liver, skeletal muscle, gut, and the cardiovascular system constitute central pillars of the metabolic network that governs global energy homeostasis. Each of these tissues contributes uniquely to MetFlex by regulating nutrient sensing, substrate uptake, storage, and energy expenditure according to physiological demands. Adipose tissue dynamically manages lipid storage and mobilization while also modulating endocrine signals through adipokines, which influence systemic metabolism and inflammation. The liver acts as a metabolic hub, integrating signals to coordinate glucose and lipid metabolism. Skeletal muscle plays a pivotal role in insulin-mediated glucose uptake and energy consumption, which directly impact systemic metabolic health. The gut microbiota and barrier integrity shape nutrient absorption and modulate systemic inflammation, while the cardiovascular system regulates nutrient delivery and vascular responses crucial for metabolic balance.

**Figure 1:**
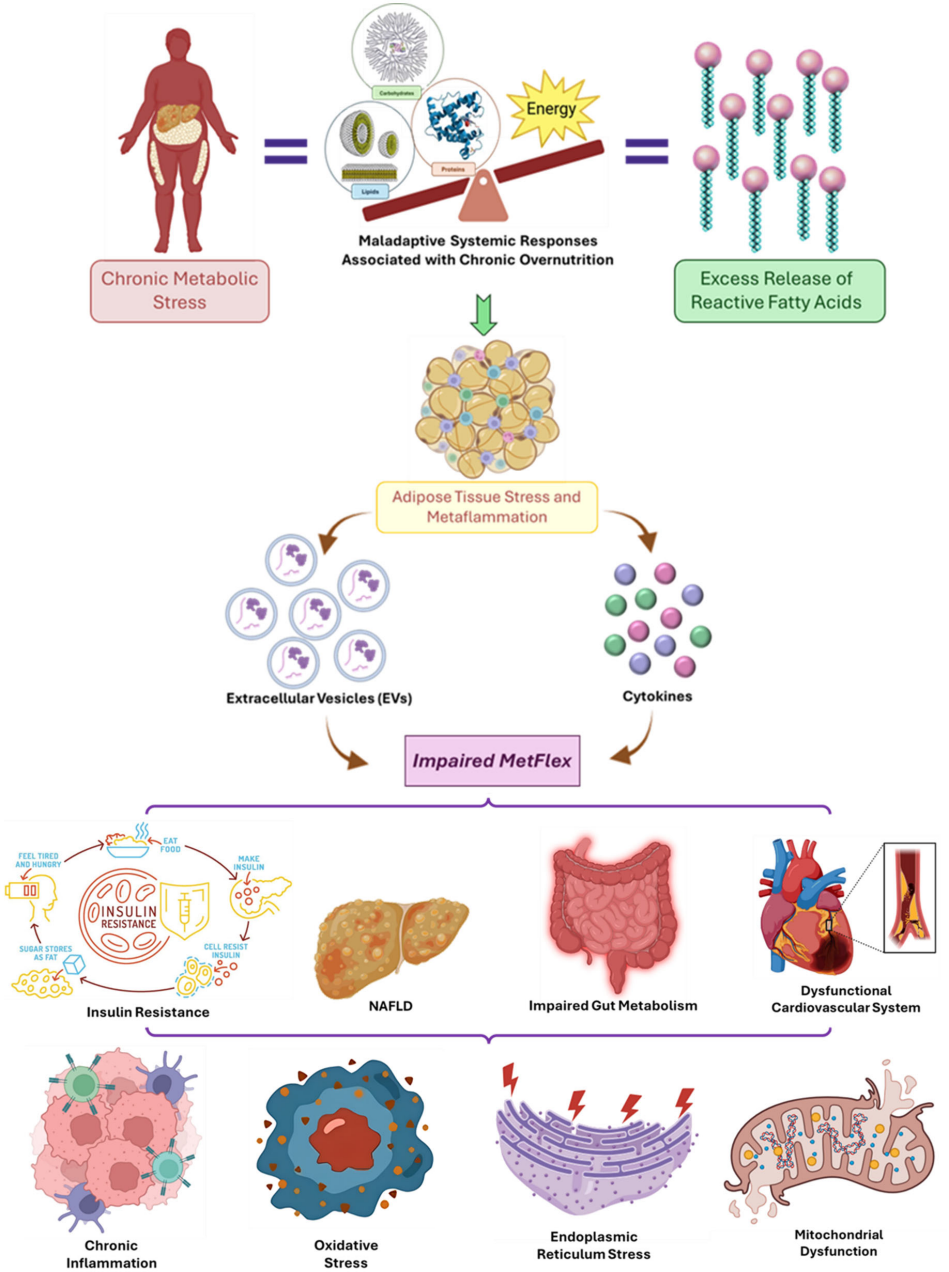
Mechanistic crosstalk between chronic metabolic stress, impaired metabolic flexibility (MetFlex), and the development of multi-system metabolic disorders.

However, growing evidence highlights how chronic metabolic stress, primarily driven by nutrient excess disrupts this flexibility (**Figure 1**), with profound consequences for whole-body metabolism (1, Bou Matar et al.).

This Research Topic, *“Exploring the Dynamics of Tissue Flexibility: Molecular Changes and Their Implications for Metabolic Disorders,”* assembles six contributions that examine tissue-specific and systemic mechanisms driving metabolic inflexibility. Collectively, these studies demonstrate the molecular underpinnings of energy dysregulation, highlight inter-organ communication, and suggest strategies to restore tissue adaptability.

## Skeletal muscle perfusion and systemic metabolic flexibility

Skeletal muscle is a primary site for glucose uptake and mitochondrial oxidative metabolism. In their research, Shoemaker et al., explored differences in muscle oxygen utilization and blood flow between sarcopenic and non-sarcopenic aged individuals during resting, post-meal, aerobic, and anaerobic exercise conditions. They found that reduced muscle oxygen saturation and impaired perfusion were closely linked to diminished systemic metabolic flexibility, reflecting how vascular and tissue-level dysfunction constrains the muscle’s ability to adapt fuel utilization to varying demands. The authors further reported that the observed metabolic inflexibility contributed to compromised mitochondrial function and increased reliance on less efficient energy substrates, which collectively drove declines in muscle strength and systemic metabolic health.

Specifically, sarcopenic individuals showed blunted increases in muscle total haemoglobin post-meal and consistently lower muscle tissue oxygen saturation (TSI) during both aerobic and anaerobic exercise compared to non-sarcopenic subjects, indicating compromised microvascular function and impaired nutrient and oxygen delivery. These vascular impairments may exacerbate metabolic inflexibility by limiting substrate delivery critical for mitochondrial efficiency and muscle energy production. Additionally, the study highlights that reduced muscle capillarization and vascular responsiveness may inhibit anabolic responses to exercise, suggesting that targeting muscle perfusion alongside nutritional and resistance training interventions could be crucial to mitigating sarcopenia progression in aging populations.

These insights emphasize the critical importance of preserving muscle blood flow and oxygen delivery through targeted nutritional and exercise interventions, particularly in aging populations where sarcopenia and metabolic dysfunction are prevalent. Maintaining vascular and metabolic health in muscle tissue is therefore essential for sustaining overall metabolic resilience and functional capacity.

## Adipose tissue dysfunction and systemic impacts

Adipose tissue is a highly dynamic organ that not only buffers excess energy, but also exerts significant endocrine and metabolic regulatory functions essential for systemic energy homeostasis. However, this delicate balance is profoundly disrupted under conditions of chronic nutrient excess, a key factor leading to impaired metabolic flexibility.

A comprehensive review by Bou Matar et al., highlights how prolonged metabolic stress triggers a cascade of cellular dysfunctions within adipocytes, leading directly to impaired metabolic flexibility. This pathology is initiated at the subcellular level with mitochondrial impairment, endoplasmic reticulum (ER) stress, and premature cellular senescence. These interconnected stressors, in turn, initiate inflammatory signalling and profoundly dysregulate the secretory profile of the tissue, marked by the suppression of protective adipokines such as adiponectin and the elevation of inflammatory mediators including TNF-α and IL-6, alongside alterations in the cargo of extracellular vesicles (EVs). The authors further detail how this dysfunction manifests structurally: the infiltration of immune cells, coupled with the development of tissue hypoxia and fibrosis, collectively restricts the capacity for new adipocyte formation and drives pathological hypertrophy. Beyond these local cellular events, the review emphasizes the influence of external modulators, such as circadian rhythm disruption and gut microbiome dysbiosis, which alter adipose metabolic gene expression and establish a complex gut-adipose axis that perpetuates systemic dysfunction. Furthermore, the contribution of brown and beige adipose depots to whole-body energy balance is discussed, particularly through thermogenic regulation mediated by key transcription factors like PGC-1α and PRDM16. The ultimate result of this comprehensive adipose dysfunction is the limitation of the tissue’s capacity to efficiently manage fatty acid storage and release, thereby severely disrupting inter-organ communication and propagating systemic insulin resistance.

Collectively, these observations establish adipose tissue as a pivotal nexus where cellular stress, inflammation, and metabolic dysregulation converge, significantly amplifying metabolic inflexibility and exemplifying the critical role of inter-organ metabolic crosstalk in metabolic disease pathogenesis.

## Lipid regulation and skeletal muscle mass

Emerging evidence indicates complex interactions between lipid metabolism and the regulation of skeletal muscle mass, challenging the traditional view of high-density lipoprotein cholesterol (HDL-C) as solely a cardioprotective biomarker. This paradigm shift is underscored by recent findings revealing a complex, sex-specific relationship between HDL-C and skeletal muscle mass (Zhang et al.). This study demonstrates that elevated HDL-C concentrations correlate with a heightened risk of reduced skeletal muscle mass in older males, a finding that was not observed in females. Such sex-dependent disparities likely reflect underlying differences in muscle fiber composition, hormonal regulation, and lipid handling mechanisms. Specifically, declining androgen levels in aging men may exacerbate muscle protein synthesis impairment, amplifying the negative impact of high HDL-C on muscle tissue, whereas in women, oestrogen decline may overshadow HDL-C effects through inflammatory pathways and mitochondrial dysfunction.

This dysregulated lipid metabolism impairs the muscle’s intrinsic capacity to efficiently switch between lipid and glucose substrates, a core aspect of metabolic flexibility. The resulting skeletal muscle dysfunction may propagate systemic metabolic inflexibility, contributing to the pathogenesis of cardiometabolic disorders such as metabolic syndrome, obesity, and chronic inflammation. These findings suggest that HDL-C, traditionally viewed as cardioprotective, may exert multifaceted roles in muscle metabolism and highlight the necessity of considering sex-specific lipid-muscle interactions in the prevention and management of sarcopenia and related metabolic diseases.

## Tissue fibrosis and remodelling

Fibrotic diseases represent a major and growing clinical challenge due to their progressive nature and profound impact on cellular and organ health and resilience, a process governed by maladaptive metabolic reprogramming and impaired metabolic flexibility. In this context, Zhao et al., provide a timely and comprehensive review examining the role of exercise as a powerful modulator of tissue fibrosis across diverse organs, including the heart, lungs, kidneys, liver, and skeletal muscle. The authors highlight that exercise exerts various effects on tissue fibrosis by modulating key pathological mechanisms, including reduced fibroblast activation, attenuated pro-fibrotic signalling pathways such as TGF-β, decreased chronic inflammation and oxidative stress, improved mitochondrial function, and regulated autophagy and apoptosis. They note that the beneficial outcomes of exercise are influenced by the type, intensity, and duration of the physical activity, with moderate and appropriately tailored exercise regimens demonstrating the most consistent antifibrotic effects across different organs. The review further emphasizes that while exercise generally improves metabolic function and tissue remodelling, excessive or unregulated exercise may exacerbate fibrosis in some contexts. By integrating these molecular and physiological insights, the authors advocate for individualized exercise prescriptions as promising non-pharmacological interventions to restore metabolic homeostasis and limit fibrotic progression, thereby potentially enhancing organ function and overall health in patients with fibrotic diseases.

## Hormonal regulation and energy substrate switching

Adipose-derived hormones are increasingly recognized as active regulators of metabolic homeostasis, prompting closer investigation of their contribution to the metabolic disturbances associated with obesity and type 2 diabetes. Specifically, the study by Liang et al., provides important insights into the adipokine asprosin, showing a clear, stepwise increase in circulating levels from normal-weight to overweight and obese individuals with T2DM. Even after adjusting for a wide set of metabolic parameters including glycaemic indices, lipid profile, blood pressure, renal function, and liver enzymes, serum asprosin remained independently associated with BMI. Notably, individuals in the highest tertile of serum asprosin had a markedly increased risk of obesity, with an odds ratio greater than 8.

Beyond its association with obesity, serum asprosin demonstrated predictive value in T2DM, with a ROC AUC of 0.770 and high specificity at a cutoff of ~332 pg/mL. Asprosin correlated positively with triglycerides, systolic blood pressure, uric acid, creatinine, and AST, and negatively with ALT, reflecting a metabolic profile consistent with altered energy metabolism and impaired substrate handling. Although causality cannot be inferred from this cross-sectional study, these findings align with mechanistic evidence that elevated asprosin may increase hepatic glucose output, stimulate appetite, and enhance inflammatory signalling, potentially limiting efficient substrate switching and highlighting its role as a biomarker and contributor to reduced metabolic flexibility in obesity and T2DM.

## Quantitative assessment of metabolic flexibility

Despite the critical importance of MetFlex for systemic health, its dynamic nature and reliance on complex, integrated physiological processes have historically made it challenging to quantify reliably and practically on a large scale, thus necessitating the development of new, functional assessment tools to bridge this gap. Recent work by Jasker et al. advances the field by introducing the MetFlex Index™ (MFI), a practical, exercise-derived marker designed to quantify metabolic flexibility through the relationship between lactate threshold power and BMI. In a cohort of 827 adults, the authors showed that MFI peaks in early adulthood, is higher in men than in women, and declines progressively with age. Lower MFI values were strongly associated with greater adiposity, higher visceral fat, elevated resting heart rate, and reduced skeletal muscle mass, whereas higher MFI corresponded to more favourable cardiovascular and body-composition profiles.

Mechanistically, the use of the first lactate threshold grounds MFI in substrate-switching physiology: earlier lactate accumulation reflects diminished fat-oxidation capacity and reduced flexibility in adapting to energetic demands. Notably, MFI remained largely independent of fasting blood metabolites, underscoring its value as a functional index rather than a biochemical surrogate. Collectively, these findings position the MetFlex Index™ as a scalable, non-invasive tool for assessing metabolic flexibility and for guiding individualized exercise strategies and cardiometabolic risk evaluation.

## Conclusion

This Research Topic provides a comprehensive overview of tissue flexibility and systemic metabolic health. By integrating findings across skeletal muscle, AT, liver, and hormonal regulation, the six contributions evidence molecular and cellular mechanisms driving metabolic inflexibility and highlight potential strategies to restore adaptive capacity. We hope this Research Topic will inspire further research that translates mechanistic insights into interventions enhancing tissue adaptability, preventing metabolic disorders, and improving overall metabolic health.
